# Pseudogenes

**DOI:** 10.1155/2012/424526

**Published:** 2012-05-07

**Authors:** Yusuf Tutar

**Affiliations:** ^1^Department of Biochemistry, Faculty of Medicine, Cumhuriyet University, 58140 Sivas, Turkey; ^2^Department of Chemistry, Faculty of Science, Cumhuriyet University, 58140 Sivas, Turkey; ^3^CUTFAM Research Center, Faculty of Medicine, Cumhuriyet University, 58140 Sivas, Turkey

## Abstract

Pseudogenes are ubiquitous and abundant in genomes. Pseudogenes were once called “genomic fossils” and treated as “junk DNA” several years. Nevertheless, it has been recognized that some pseudogenes play essential roles in gene regulation of their parent genes, and many pseudogenes are transcribed into RNA. Pseudogene transcripts may also form small interfering RNA or decrease cellular miRNA concentration. Thus, pseudogenes regulate tumor suppressors and oncogenes. Their essential functions draw the attention of our research group in my current work on heat shock protein 90: a chaperone of oncogenes. The paper reviews our current knowledge on pseudogenes and evaluates preliminary results of the chaperone data. Current efforts to understand pseudogenes interactions help to understand the functions of a genome.

## 1. History of Pseudogenes

 Sequencing human genome brought several debates about noncoding sequences. So what is the role of the noncoding parts since protein coding exons compromise only around two percent of the whole genome sequence? The noncoding regions are transposable elements, structural variants, segmental duplications, simple and tandem repeats, conserved noncoding elements, functional noncoding RNAs, regulatory elements, and pseudogenes [1]. Annotation of these noncoding regions through functional genomics and sequence analysis helps our understanding of genomics.

Noncoding regions of human genome in general were thought to be nonfunctional and “junk,” or of no purpose DNA. Nowadays, scientists are conceding that junk DNA terminology is far from true since recent studies indicate that they have some regulatory roles. This work focuses on pseudogenes of junk DNA. Pseudogenes are gene copies that have coding-sequence deficiencies like frameshifts and premature stop codons but resemble functional genes.

The first pseudogene was reported for 5S DNA of *Xenopus laevis*, coding for oocyte-type 5S RNA, in 1977, and several pseudogenes have been reported and described for a variety of species including plants, insects, and bacteria [2, 3].

Currently, approximately twenty thousand pseudogenes are estimated which is comparable to the number of protein-coding genes (around 27000) in human [4]. Current knowledge of these genes remains poorly understood, and many sequences once believed defunct are in fact functional RNA genes and play roles in gene silencing either by forming siRNAs or by changing mRNA levels of functional protein-coding gene [5]. Several studies focused on the pseudogene population and their regulatory roles as the function of more pseudogenes is being uncovered. It is interesting to compare and contrast genes from a variety of organisms to determine their adaptation for survival. Pseudogenes provide a record of all changes in the genome of a particular organism.

## 2. Types of Pseudogenes

Pseudogenes can be categorized in two forms: unprocessed and processed. Unprocessed pseudogenes can also be subcategorized as unitary and duplicated [3, 6].

Pseudogenes originate from decay of genes that originated from duplication through evolution. The decays include point mutations, insertions, deletions, misplaced stop codons, or frameshifts of a gene. The decay may occur during duplication, and these disablements may cause loss of a gene function. Loss of productivity, expression of RNA or protein coding ability, results in the production of unprocessed pseudogenes. A unique subfamily of unprocessed pseudogenes are described by Zhang et al. Formation of nonduplicated unprocessed pseudogenes is named “unitary” pseudogenes [7]. In unitary type of pseudogenes, a single copy parent gene becomes nonfunctional. Unprocessed and duplicated pseudogenes keep their intron-exon structure. Processed pseudogenes are formed through retrotransposition. Retrotransposition occurs by reintegration of a cDNA, a reverse transcribed mRNA transcript, into the genome at a new location. The double-stranded sequences of processed pseudogenes are generated from single-strand RNA by RNA polymerase II rather than the RNA polymerase III. Therefore, processed pseudogenes lack introns, 5′ promoter sequence, and have flanking direct repeats and 3′ polyadenylation tag. The overall distribution of most pseudogenes is completely random, duplicated, and processed pseudogenes are found in the same or on different chromosome of their parent genes.

Duplication of DNA segments explains the generation of gene families from a common ancestral gene. The dynamic nature of genome cause changes in its composition with time.

## 3. Why Do Organisms Keep Pseudogenes?

Why organisms maintain pseudogenes and pay a cost of energy? Replication of these genes over generations is a disadvantageous biochemical process. Why would not natural selection remove these costly DNA segments? What is the potential benefit to keep non-protein-coding sequences? Why are highly expressed genes more likely to produce pseudogenes? Do the pseudogenes accumulate all kinds of mutation including deleterious ones to protect the functional genes?

Gene duplications make functional divergence and generate new genes [8]. Unprocessed pseudogenes have introns and regulatory sequences, and their expression is crippled by stop codons. The extra copies of functional genes accumulate mutations, and this maintains original gene functional. Gene duplication may give rise to a new gene with completely different function. Recent papers indicate that some pseudogenes exhibit functional roles such as gene expression and gene regulation. Genetic code of an organism can be duplicated by copying errors, and these duplicated genes would be passed down from generation to generation. Since pseudogenes accumulate mutations over years, number of mutations of these so-called fossil molecules provide an estimate of their age. Further studies on the pseudogene evolution may give insight into their mechanism of action.

## 4. Conservation of Mutations

Various pseudogenes have certain conserved mutations in different species. Conservation of pseudogenes was explored in human, chimpanzee, mouse, rat, dog, and cow. Pseudogenes from different species have point mutations and even specific types of mutations at certain gene locations. The shared mutations in different organisms are thought to depend on common descent or evolutionary ancestry [9].

The locus of insertion of a pseudogene determines its evolution. Deleterious insertions will be selected, and the pseudogene will be lost; however, pseudogenes with other nondeleterious mutations persist and evolve over time. Processed pseudogenes evolve more rapidly than their functional paralogs and undergo genetic drift with random mutations, deletions, and insertions. Established pseudogenes can pass to next generation and may partially be duplicated to give a second pseudogene. Thus, pseudogenes provide a powerful tool for phylogenetic studies to investigate genome evolution.

## 5. Mechanism of Action

What is the potential benefit to retain pseudogene? There is evidence that interaction of pseudogenes with their functional genes regulates different biochemical processes in cells. The pair of genes may influence expression of a functional gene mRNA overexpression of a pluripotency-associated transcription factor; *Oct4 *pseudogene transcript inhibits cell differentiation. And knockdown of *Oct4 *pseudogene RNA antisense increases the levels of *Oct4 *and its two pseudogenes. Examples of these types of experiments provide evidence that antisense pseudogene transcripts combine with sense genic transcripts, and this regulates functional gene expression level [3, 4].

Small interfering RNA (siRNA) also regulates gene expression. It was shown in mouse oocytes that folded pseudogenes transcripts form hairpin structures to form siRNAs, and these siRNAs repress gene expression. Experiments with loss of siRNA producer protein, Dicer, cause a decrease in the levels of pseudogene-derived siRNAs and an increase of coding gene mRNAs. The experiments support siRNA-dependent regulation.

One last potential mechanism of pseudogene function is interfering with factors that regulate mRNA stability. *Trans*-acting molecules interaction with *cis*-acting sequences of mRNA stabilizes mRNA molecule. Pseudogenes which have *cis-*acting sequences similar to functional gene compete for *trans*-acting molecules. This competition decreases mRNA stability and expression [3, 6].

Micro RNAs (miRNA) affect mRNA stability through pairing mainly with 3′ untranslated region of mRNA. miRNAs cause degradation of the mRNA and decrease levels of expression. *PTEN *is a tumor suppressor and maintaining certain level of PTEN protein prevents oncogenesis. Coupled miRNAs coregulate both the gene *PTEN *and pseudogene *PTENP1*. *PTENP1 *pseudogene binds miRNA and reduces cellular concentration of miRNA. This allows *PTEN *to escape from miRNA repression regulation [3].

The above-mentioned evidence shows that some pseudogenes play essential roles in translational interference or siRNA generation and that can silence a gene. Alternatively, protein coding mRNA and their corresponding pseudogenes can compete for stabilizing factors and/or miRNAs and this alters protein coding mRNA expression levels.

## 6. Identification of Pseudogenes

High-sequence similarity between pseudogenes and their functional partners poses a challenge to scientists with frequent misidentification. It is not possible to quantify the number of pseudogenes within a genome even until the genome is completely sequenced. Genomes include several paralogous pseudogenes, and many genes do not have pseudogenes.

Pseudogenes can be located anywhere within a genome, and retrotransposition of processed pseudogenes causes them to be clustered adjacent to their paralogous functional gene or can be inserted into a different chromosome. Pseudogenes originate from mitochondrial DNA can be inserted in nuclear DNA, and this makes pseudogene identification difficult.

The complexity of the identification of pseudogenes can be overcome by *in silico *analysis. The problematic identification of pseudogenes is simpler by using a homology-based whole genome identification approach. It is critical to identify pseudogenes to understand genome annotation and disease-related molecular mechanism. Identification of pseudogenes is an ongoing effort, and there are several groups continuously working on identification of pseudogenes. There are different methods developed by independent groups to identify pseudogenes such as REGEXP, PseudoPipe, PseudoFinder, RetroFinder, and GIS-PET [10, 11].

## 7. Evolutionary Fate of Pseudogenes

Pseudogene sequences resemble to their parental gene. Pseudogenes have been considered as evolutionary relics however as we understand the mechanism of new gene generation this phenomenon changes. New gene generation mechanism involves at molecular level at germ line [12–14]. New gene generation follows a variety of mechanism: gene duplication, transposable element protein domestication, gene fusion, gene fission, lateral gene transfer, and de novo origination. Details of new gene generation and evolution can be found in recent excellent reviews [12–14]. Once a new gene is generated, the gene serves as starting point for evolution. The main creative force in evolution is gene duplications as proposed by Dr. Susumu Ohno [15]. Gene duplication produces copies of a gene, and usually one copy maintains gene function while the other(s) may gain new functions. Alternatively, duplication may result with gene loss or pseudogenization. The abundance of pseudogenes generally depends on rates of gene duplication and loss. Comparative analysis of fully sequenced genomes shows that the size and complement of gene families are dynamic than expected. Thus, this supports the idea of gene duplication which is the principle force in evolution. Genetic divergence may also be explained by copy number variation [16].

To define the birth and the death of gene families and to define ambiguous boundary between genes and pseudogenes are challenging [16, 17]. Nonfunctionality for pseudogenes can be difficult to define. This ambiguity was first appeared by nitric oxide synthase (NOS) from *Lymnaea stagnalis*, a snail. In certain neurons NOS pseudogene acts as antisense RNA and decreases mRNA transcript expression through hybridization to NOS mRNA. The NOS pseudogene has defects and cannot code for a protein like its parental gene [17].

Zheng and Gerstein classified genes and pseudogenes by defining living gene, ghost pseudogene, and dead pseudogene [17]. This classification clearly relates functionality to define the birth and the death of gene families. In this context living gene is described as protein coding genes and dead pseudogene is described as not transcribed and evolves neutrally. This nonfunctional dead pseudogene was categorized to two classes depending on genetic defects as nondisabled and disabled pseudogenes [17].

Intermediate functionality between living gene and dead pseudogene is described as ghost pseudogene. Ghost pseudogenes are further divided into three categories: exapted pseudogene, piggy-back pseudogene, and dying pseudogene.

However, this categorization is still not sufficient to define functionality since parts of several pseudogene transcripts can fuse to form chimeric RNAs.

In spite of these dilemmas, pseudogenes are important for comparative genomics since they provide records of ancient genes. These ancient genes are important for evolutionary and comparative genomics. Therefore, identification of pseudogenes is important to determine the rate and age of gene duplication. Neutral character of pseudogene regions helps us to determine different forms and rates of sequences and evolution within the sequence of an organism and among different organisms.

Only few sequencing projects and database include pseudogenes. Estimates of pseudogene numbers rely on extrapolation. A study on human chromosome 21 and 22 indicated presence of 393 total pseudogenes, and this was extrapolated to an approximate 20 000 human pseudogenes [18]. A different method determines an estimate between 23000 and 33000 processed pseudogenes in humans. The extrapolation assumes 75000 to 10000 human genes; however the total number of processed pseudogenes decreases to 9000–11000 when the total human genes are assumed to be 30000–35000 [18, 19].

All extrapolation methods predict the number of processed pseudogenes as one third of the total number of human genes. Several studies are underway to determine the number of pseudogenes with different implementations to the methodology [19, 20].

The evolution of duplicated genes and pseudogenization process led scientist to different models: mutational and epigenetic complementation. According to mutational model degenerative mutations are protected from degradation and independent regulatory element controls specific expression. The epigenetic complementation model is silencing of duplicates by methylation and by RNA inhibition mediated silencing. This model assumes that gene duplicate is different and epigenetic mechanism will not control the duplicate gene [21].

## 8. Future Perspectives

How does current knowledge on pseudogenes consist with experimental data? Cell miner (http://discover.nci.nih.gov/cellminer/) provides a relational database for NCI-60 cancer cell lines. Cell miner is a database which will be open to public soon. The cell lines were profiled at the DNA, RNA, protein, and pharmacological levels by using microarray-technology-based data [22].

Querying of molecular information on all known human heat shock genes provides pseudogene and miRNA-dependent regulation. The pseudogenes involved in the mechanism are mostly Hsp90s. Hsp90 is a highly expressed gene, two percent of the total protein expressed, and has multiple retrotransposed pseudogenes. Microarray data indicates that Hsp90 pseudogenes (HSP90AA1 and HSP90AA2) along with several microRNAs may interfere with Hsp90 genes (Hsp90AB1 and HSP90B1) at transcription level. We are performing experiments for further evidence in my laboratory.

As a conclusion, pseudogenes are essential parts of gene regulation. Understanding the mechanism of pseudogene action may help researchers to solve several essential biochemical pathways. Human Hsp90 chaperone has numerous client proteins. There are key nodal signaling proteins, and inhibition of Hsp90 may stop or weaken a cancer safety net. Hsp90 pseudogenes might be functional and participate in gene expression and molecular mechanism of the interactions, and this may help understanding the roles of each factor and provide an in-depth description of the many signaling nodes regulated by pseudogenes.
